# Supplementary material for: Effects of Hyperoxia on the Refraction in Murine Neonatal and Adult Models
Source: Int J Mol Sci. 2019 Nov 29;20(23):6014. doi: 10.3390/ijms20236014 (PMC6928741; doi:10.3390/ijms20236014)
Supplement: Supplementary file 1 [file ijms-20-06014-s001.pdf]

Supplementary Information for

Effects of hyperoxia on the refraction in murine neonatal and adult models

Table of Contents:

Supplementary Table 1

Supplementary Table 2

Supplementary Table 1. Change of each ocular component from P30 to P47 in neonatal mice.

		normoxia	hyperoxia	*P* value
refraction (D)	P30	+0.47±2.2	-5.82±3.46	<0.001
	P47	+4.11±2.02	-6.56±5.89	<0.001
	Change	3.04±2.22	-0.74±5.96	0.097
AL (mm)	P30	3.18±0.03	3.16±0.03	0.057
	P47	3.31±0.05	3.31±0.04	0.852
	Change	0.13±0.03	0.15±0.03	0.106
CR (mm)	P30	1.35±0.04	1.34±0.02	0.626
	P47	1.45±0.02	1.43±0.02	0.131
	Change	0.11±0.03	0.09±0.03	0.234
CCT (μm)	P30	97.13±7.99	94.00±6.26	0.374
	P47	94.75±0.96	99.64±2.98	<0.001
	Change	-3.00±5.89	5.64±7.39	0.052
ACD (mm)	P30	0.33±0.02	0.32±0.01	0.492
	P47	0.37±0.02	0.37±0.02	0.866
	Change	0.03±0.01	0.05±0.02	0.044
LT (mm)	P30	1.80±0.01	1.77±0.02	0.003
	P47	1.96±0.02	193±0.03	0.033
	Change	0.16±0.01	0.16±0.02	0.797

P30: 30 days old, P47: 47days old, D: Diopter, AL: Axial

length, CR: corneal radius, CCT: central corneal thickness, ACD: anterior chamber depth, LT: lens thickness

Supplementary Table 2. Change of each ocular component from P5w to P26w in adult mice.

		normoxia	hyperoxia	*P* value		Period from P5w	normoxia	hyperoxia	*P* value
refraction	P5w	+6.04±1.58	+4.46±1.34	0.090	Change				
(D)	P7w	+10.71±4.05	-6.78±3.06	<0.001	in	2w	4.66±4.24	-11.24±3.41	<0.001
	P12w	+7.52±2.54	-7.20±4.09	<0.001	refraction	7w	1.48±3.12	-11.69±5.09	<0.001
	P19w	+6.25±3.65	-0.79±6.10	0.042	from	14w	0.21±3.04	-5.25±6.78	0.115
	P26w	+3.29±5.17	-9.72±2.89	<0.001	P5w (D)	21w	-2.75±5.36	-14.19±3.57	0.002
AL (mm)	P5w	3.21±0.02	3.21±0.05	0.869	Change				
	P7w	3.31±0.02	3.33±0.04	0.143	in AL	2w	0.10±0.02	0.12±0.03	0.149
	P12w	3.43±0.01	3.44±0.03	0.545	from	7w	0.23±0.01	0.23±0.05	0.822
	P19w	3.52±0.03	3.52±0.05	0.799	P5w	14w	0.31±0.02	0.31±0.05	0.917
	P26w	3.57±0.03	3.59±0.04	0.351	(mm)	21w	0.36±0.05	0.37±0.03	0.475
CR (mm)	P5w	1.36±0.05	1.38±0.02	0.446	Change				
	P7w	1.45±0.04	1.43±0.02	0.279	in CR	2w	0.09±0.04	0.05±0.03	0.070
	P12w	1.50±0.03	1.44±0.03	0.003	from	7w	0.14±0.05	0.06±0.04	0.016

	P19w	1.49±0.04	1.49±0.03	0.832	P5w (mm)	14w	0.13±0.00	0.11±0.05	0.535
CCT (μm)	P5w	98.50±1.97	99.83±4.88	0.549	Change				
	P7w	100.33±3.88	110.17±8.09	0.023	in CCT	2w	1.83±4.17	10.33±6.83	0.032
	P12w	99.50±6.22	110.67±3.33	0.003	from	7w	1.00±7.43	10.83±6.56	0.035
	P19w	103.00±9.27	102.67±2.00	0.972	P5w	14w	4.50±1.00	2.83±19.02	0.854
	P26w	94.17±13.85	106.17±14.74	0.177	(μm)	21w	-4.33±12.8	2.83±15.19	0.218
ACD (mm)	P5w	0.33±0.01	0.34±0.01	0.496	Change				
	P7w	0.35±0.01	0.37±0.01	0.026	in ACD	2w	0.02±0.01	0.03±0.01	0.032
	P12w	0.39±0.01	0.39±0.01	0.982	from	7w	0.06±0.01	0.06±0.02	0.586
	P19w	0.39±0.02	0.37±0.04	0.255	P5w	14w	0.06±0.02	0.03±0.06	0.261
	P26w	0.41±0.01	0.40±0.04	0.642	(mm)	21w	0.07±0.01	0.06±0.04	0.459
LT (mm)	P5w	1.85±0.01	1.85±0.04	0.876	Change				
	P7w	1.96±0.02	1.99±0.05	0.292	in LT	2w	0.11±0.02	0.14±0.02	0.044
	P12w	2.11±0.01	2.11±0.02	0.673	from	7w	0.25±0.01	0.26±0.02	0.463
	P19w	2.22±0.01	2.22±0.03	0.779	P5w	14w	0.36±0.01	0.37±0.03	0.634
	P26w	2.27±0.02	2.27±0.02	0.715	(mm)	21w	0.42±0.02	0.42±0.03	0.857

P5w: 5 weeks old, P7w: 7 weeks old, P12w: 12 weeks old, P19w: 19 weeks old, P26w: 26 weeks old, w: weeks, D: Diopter, AL: Axial length, CR: corneal radius, CCT: central corneal thickness, ACD: anterior chamber depth, LT: lens thickness
